# Heteroatom-doped porous carbons derived from moxa floss of different storage years for supercapacitors†

**DOI:** 10.1039/c8ra01672k

**Published:** 2018-05-03

**Authors:** Xuelin Zhang, Qingyuan Niu, Yaqing Guo, Xiyan Gao, Kezheng Gao

**Affiliations:** College of Acupuncture-Moxibustion and Tuina, Henan University of Traditional Chinese Medicine Zhengzhou 450046 China gaoxiyan@yeah.net +86-371-65934802; State Laboratory of Surface and Interface Science and Technology, School of Material and Chemical Engineering, Zhengzhou University of Light Industry Zhengzhou 450002 China gaokezheng@126.com

## Abstract

Two novel carbons (MCs) derived from moxa floss of different storage years have been prepared by two low-cost and facile approaches, which are hydrothermal carbonization at a low temperature (200 °C) and direct pyrolysis at a moderate temperature (500 °C) followed by potassium hydroxide (KOH) activation strategy at a high temperature (800 °C), respectively. The physicochemical properties of MCs are investigated by Raman spectra, X-ray diffraction (XRD), X-ray photoelectron spectroscopy (XPS), field-emission scanning electron microscopy (FESEM), transmission electron microscopy (TEM), and nitrogen adsorption–desorption isotherms. Results show that MCs derived from moxa floss of different storage years by two facile approaches possess different morphologies: MCs by hydrothermal carbonization (denoted as MC-1, MC-2 and MC-3) exhibit porous nanosheet structures, the highest specific surface area is about 1788.6 m^2^ g^−1^, and the largest total pore volumes is around 0.8170 cm^3^ g^−1^, while MCs by direct pyrolysis (denoted as MC-4, MC-5 and MC-6) have basically blocky and rod-like morphologies, the highest specific surface area is about 1628.0 m^2^ g^−1^, and the largest total pore volume is around 0.7058 cm^3^ g^−1^. However, despite the different morphologies, all MCs possess a similar hierarchical porous structure, numerous heteroatom groups and good electrical conductivity. Therefore, these low-cost, biomass-derived porous carbons with promising capacitive performance are used for supercapacitors application with high performance, for example, the as-assembled supercapacitor based on MC-5 exhibits a high specific capacitance of 288.3 F g^−1^ at 0.25 A g^−1^, an excellent rate performance of 243.5 F g^−1^ even at 30 A g^−1^ with 84.5% capacitance retention of its initial specific capacitance, and an outstanding long-term cycling stability with 98.7% capacitance retention after 10 000 cycles at 5 A g^−1^. Furthermore, the maximum energy density for these supercapacitors with an aqueous electrolyte in a two-electrode system is about 10.0 W h kg^−1^ at a power density of 70.3 W kg^−1^. Therefore, this work opens up a whole new field for the applications of moxa floss and this novel concept of moxa floss use is an extremely promising strategy for developing high-performance carbons with porous structures and heteroatom-doping from renewable sources.

## Abstract 2

Two novel carbons (MCs) derived from moxa floss of different storage years have been prepared by two low-cost and facile approaches, which are hydrothermal carbonization and direct pyrolysis followed by KOH activation strategy, respectively.

## Graphical abstract

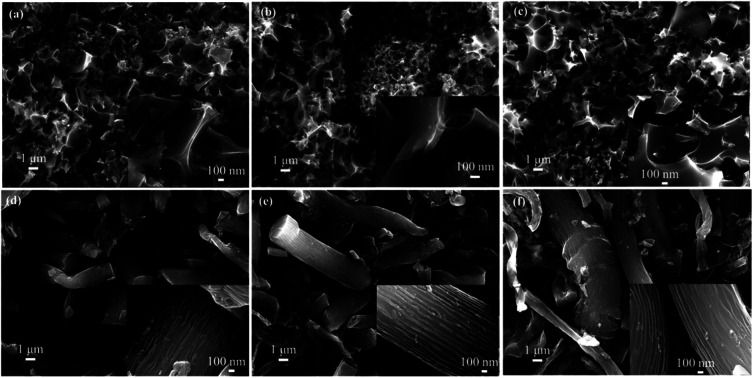

## 1. Introduction

Supercapacitors, owing to their key advantages of rapid charge–discharge rates, long-term cycling life, superior power densities, excellent safety, minimal maintenance costs, and environmental friendliness, have widespread applications in portable devices, hybrid electric vehicles, power tools, and energy backup systems.^1–8^ Electrical double-layer capacitors (EDLCs), which are the most widespread type of supercapacitors, store the electrostatical charges through reversible adsorption of ions at the interface of electrode and porous electrodes.^9–12^ A wide variety of carbon materials, including activated carbons (ACs), carbon nanofibers, carbon nanotubes, graphene, carbon aerogel, have been intensively utilized as electrode materials in EDLCs.^13–22^ Carbon materials with the merits of high specific surface area (SSA), controlled hierarchical porosity, heteroatom doping, excellent electrical conductivity, high chemical stability, lightweight and low cost, have drawn significant research attention.^23,24^ In general, the high specific surface areas and micropores ensure a high energy density, mesopores guarantee a high power density. The morphologies and heteroatom doping of carbon materials are strongly related to their electrical conductivity. However, the electrical conductivity and chemical stability of carbon materials greatly influence the power density and cycling stability of carbon-based supercapacitors.^25–27^ Therefore, there is in dire need to fabricate carbon materials with enhanced features.

For years, carbon materials have been successfully produced from fossil materials,^28^ macromolecules,^14^ and biomass materials.^9,29^ Compared to traditional carbon precursors, biomass materials, such as *Eulaliopsis binata*,^30^ tobacco rods,^27^ banana peel,^31^ silk,^32^ rice husk,^33^ tealeaves,^34^ leaf,^35^*Auricularia*,^36^ coconut shell,^37^*etc.* have attracted tremendous interest owning to their large availability, low cost, renewability, and environmental friendliness. Generally, biomass-based carbons are prepared by a two-step procedure, in which carbon precursors are directly pyrolyzed at the temperature range of 600–900 °C in an inert atmosphere,^38^ or hydrothermally carbonized at a relatively low temperature,^27^ followed by physical or chemical activation at elevated temperature.^30^ In recent years, KOH activation has achieved giant success in the generation of carbon materials with the features of good electrical conductivity, abundant porous structure, heteroatom doping, ultrahigh specific surface areas and excellent physicochemical stability.^27,30,39^ Therefore, most of these carbon-based supercapacitors offer a large number of attractive attributes, such as fast charge–discharge rates, high thermal stability, good operational safety, excellent cycling stability, high power capability.^26,40^ There is still an urgent need to explore new biomass precursors for the production of carbon materials.

Moxa floss is processed from the dried leaves of *Artemisia argyi* Levl. Et Vant and used for moxibustion in Traditional Chinese Medicine.^41,42^*Artemisia argyi* is a common herbaceous perennial aromatic grass with a creeping rhizome belonged to the family compositae and widely distributed in Northeastern Asia, such as China, Mongolia, Japan, Korea and the Russian Far East regions.^43^ It is annually renewable, available in abundance and naturalized in dry and semiarid habitats, such as dry mountain slopes, steep river banks, coastal scrub, wasteland and along road and railway verges, which is beneficial to protect soil and water.^44^ The chemical composition of *Artemisia argyi* includes polysaccharides (natural cellulose), flavonoids, essential oil, triterpenoids, sesquiterpenoids, steroids, nitrogen compounds (amine, protein, amide, alkaloids, urea, *etc.*), carboxylic ester, and inorganics (sodium, calcium, potassium, magnesium, *etc.*).^45,46^ The leaves of *Artemisis argyi* are usually stored for different years before processing into moxa floss. During storage, a number of physicochemical and physiological changes occur. The aged and dried *Artemisia argyi* leaves are then subjected to several rounds of pulverization and sifting.^47,48^ Considering that *Artemisia argyi* leaves are abundant, cheap, and renewable biological resources,^49^ they should be a kind of promising carbon precursor for the production of carbon materials. To the best of our knowledge, there is no report on the use of *Artemisia argyi* leaves of different storage years as biomass precursor to synthesize carbons. Obviously, pursuing new potential applications of these leaves is a meaningful choice for enriching the use of *Artemisia argyi*.

In this study, we presented a systematic investigation on the differences in electrochemical properties of the biomass-derived carbons by two facile approaches using moxa floss of different storage years as the carbon precursor. The moxa floss of different storage years was directly subjected to hydrothermal carbonization at a low temperature (200 °C) (or pyrolysis at a moderate temperature (500 °C)) followed by potassium hydroxide (KOH) activation at a high temperature (800 °C), giving moxa floss-derived porous carbons (MCs). These resulting carbons possess different morphologies but exhibit high specific surface area up to 1788.6 m^2^ g^−1^, hierarchical porous structure coexisting of micro-, meso-, and macro-pores, numerous heteroatom (O and N) functional groups, long-term cycling stability as well as excellent capacitive performance, for example, the as-assembled supercapacitor based on MC-5 exhibits a high specific capacitance of 288.3 F g^−1^ at 0.25 A g^−1^, an excellent rate performance of 243.5 F g^−1^ even at 30 A g^−1^ with 84.5% capacitance retention of its initial specific capacitance, and an outstanding long-term cycling stability with 98.7% capacitance retention after 10 000 cycles at 5 A g^−1^. Therefore, using moax floss as carbon source to prepare biomass-derived carbons for high-power supercapacitors is an extremely promising strategy for developing low-cost and high performance electrode materials and pursuing new application of *Artemisia argyi*.

## 2. Experimental

### 2.1 Materials

Moxa floss were obtained from a moxa floss production factory in Nanyang, Henan Province, China, which included different storage years (0, 3, and 5) with the production ratio of 3 : 1. Storage year refers to the number of years that the dried mugwort leaves were stored before processing into moxa floss, while production ratio refers to the weight of the starting material (dried mugwort leaves) to the weight of the finished product (moxa floss) after processing, for example the ratio 3 : 1 means 3 kg of dried mugwort leaves is processed into 1 kg of moxa floss. The moxa floss was repeated rinsed with distilled water for about three days and dried. Three types of moxa floss were denoted as MF-1 (0 storage year and 3 : 1 ratio), MF-2 (3 storage years and 3 : 1 ratio) and MF-3 (5 storages year and 3 : 1 ratio), respectively. All of the other chemicals were analytical grade and obtained from commercial suppliers without further treatment.

### 2.2 Preparation of moxa floss-derived carbons (MCs)

#### 2.2.1 By hydrothermal carbonization-KOH activation strategy

Typically, the dried moxa floss (4 g) and distilled water (60 mL) were added into a 100 mL Teflon lined stainless autoclave. The autoclave was kept at 200 °C for 20 h and then cooled to room temperature naturally. The resulting solid product was collected by filtration and washed abundantly with distilled water, and then dried at 100 °C overnight in a conventional oven. After that, the solid product was impregnated with KOH in an aqueous solution (the weight ratio of the solid product to KOH was 1 : 3) and the mixture was stirred for 2 h at room temperature, which was followed by evaporation step at 100 °C in a conventional oven. The dried mixture was ground to powder in an agate mortar, and then heated in a tubular furnace at 800 °C for 1 h under a nitrogen gas flow with the heating ramp rate of 4 °C min^−1^. After cooling down to room temperature, the activated product (MC) was washed several times with 1 mol L^−1^ HCl solution to remove inorganic salts, and then with distilled water until neutral pH was achieved. Finally, MC was dried in a conventional oven at 100 °C overnight, and then ground to powder in an agate mortar. MCs derived from different moxa floss by hydrothermal carbonization were denoted as MC-1 (MF-1), MC-2 (MF-2), MC-3 (MF-3), respectively.

#### 2.2.2 By direct pyrolysis-KOH activation strategy

Typically, the dried moxa floss (7 g) was directly pyrolyzed in a tubular furnace under N_2_ flow for 2 h. The final temperature and heating ramp rate of the furnace were 500 °C and 4 °C min^−1^. After cooling down to room temperature, the resulting pre-carbonized product was impregnated with KOH in an aqueous solution (the weight ratio of the solid product to KOH was 1 : 3) and the mixture was stirred for 2 h at room temperature, which was followed by evaporation step at 100 °C in a conventional oven. The dried mixture was ground to powder in an agate mortar, and then heated in a tubular furnace at 800 °C for 1 h under a nitrogen gas flow with the heating ramp rate of 4 °C min^−1^. After cooling down to room temperature, the activated product (MC) was washed several times with 1 mol L^−1^ HCl solution to remove inorganic salts, and then with distilled water until neutral pH was achieved. Finally, MC was dried in a conventional oven at 100 °C overnight, and then ground to powder in an agate mortar. MCs derived from different moxa floss by direct pyrolysis were denoted as MC-4 (MF-1), MC-5 (MF-2), MC-6 (MF-3), respectively.

### 2.3 Structural characterization

The morphologies of the obtained porous carbons (MCs) were observed with field-emission scanning electron microscope (FESEM, JSM-7001F) and transmission electron microscopy (TEM, JEM-2100). The structure of MC was investigated by X-ray diffraction (XRD, Bruker D8 ADVANCE; Cu Kα radiation). Raman spectra were collected on a HORIBA Scientific LabRAM HR Evolution Raman Spectrometer system with 514 nm laser. The surface elemental characterization of MC was performed by X-ray photoelectron spectroscopy (XPS, ESCALAB 250Xi) with a monochromatic Al Kα source. All binding energies were referenced to the C1s neutral carbon peak at 284.8 eV. The textural properties were characterized by N_2_ adsorption analyses (BELSORP-Mini II) at 77 K. The samples were outgassed at 150 °C for 12 h under a vacuum. The specific surface area (SSA) was obtained by using the Brunauer–Emmett–Teller (BET) model. The pore size distribution (PSD) was calculated from the adsorption branches of the isotherms using the Barrett–Joyner–Halenda (BJH) model.

### 2.4 Supercapacitors preparation and electrochemical measurements

The electrochemical performances of MC were evaluated in a symmetrical two-electrode system (supercapacitors device). The working electrodes were typically fabricated by mixing MC as the active material (85 wt%), acetylene black (10 wt%), and polytetrafluoroethylene (PTFE, 5 wt%) binder in ethanol. The obtained electrode materials were coated onto the nickel foam current collectors (1 cm × 1 cm) with a spatula. The pre-made electrodes were dried at 100 °C for 12 h in a vacuum oven. After drying, the electrodes were pressed under a pressure of 10 MPa for 1 min. The two nearly identical (by weight and size) MC electrodes were separated using a polypropylene membrane separator soaked with electrolytes (6 mol L^−1^ KOH or EMIMBF_4_) in a 2016 stainless steel coin cell. In the case of ionic liquid electrolyte, the preparation process of working electrodes was the same as that for the aqueous electrolyte, but the assembly process was carried out in a nitrogen-filled glove box. All the electrochemical measurements except for cycling stability tests were carried out on a CHI 660E electrochemical workstation. The cycling stability tests were studied on a Land cell tester. The electrochemical properties of MC were characterized by cyclic voltammetry (CV), galvanostatic charge–discharge (GCD), and electrochemical impedance spectroscopy (EIS). CV tests were carried out between 0 and 1.0 V for the aqueous electrolyte and between 0 and 3.5 V for the ionic liquid electrolyte. GCD tests were performed at different current density varying from 0.25 to 30 A g^−1^ in the same potential range as the CV test. EIS tests were carried out with the amplitude of 5 mV in the frequency range of 10^5^ to 10^−3^ Hz referring to open circuit potential. The specific capacitance (*C*_s_) of the two-electrode device based on GCD was calculated according to the equation:^30^*C*_s_ = 4*I*/(*m* × Δ*V*/Δ*t*), where *I* (A) is the constant charge–discharge current, *m* (g) is the total mass of active materials on the two electrodes for MC supercapacitor, Δ*t* (s) is the discharge time, Δ*V* (V) is the voltage change excluding the IR drop during the discharge process, and Δ*V*/Δ*t* is calculated from the slope of the discharge curve after the voltage drop. The energy density (*E*, W h kg^−1^) and power density (*P*, W kg^−1^) of MC supercapacitors were calculated using the equations:^10,40,50^*E* = *C*_s_ × *V*^2^/(8 × 3.6), *P* = *E*/Δ*t*, where *C*_s_ (F g^−1^) is the specific capacitance of the MC supercapacitors, *V* (V) is the voltage after IR drop, and Δ*t* (s) is the discharge time.

## 3. Results and discussion

### 3.1 Morphologies and structures of MC

**Fig. 1:**
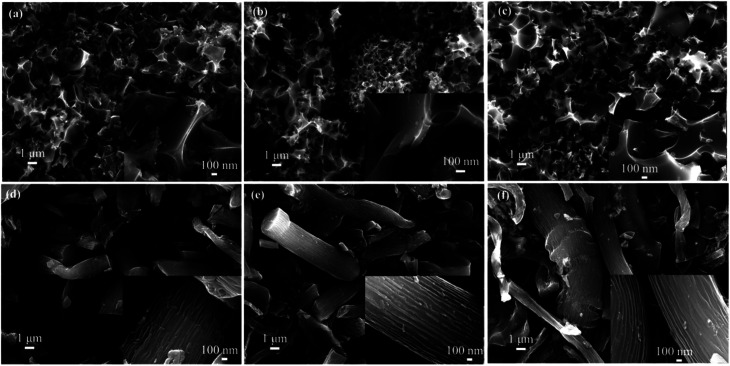
FESEM images of MC: (a) MC-1, (b) MC-2, (c) MC-3, (d) MC-4, (e) MC-5, and (f) MC-6. The insets are FESEM images under high magnification.

The morphologies of MC were characterized by FESEM observations (Fig. 1). Regardless of moxa floss of different storage years as carbon precursors, three samples of MC (MC-1, MC-2 and MC-3) fabricated by hydrothermal carbonization exhibit the porous nanosheet structures, but three samples of MC (MC-4, MC-5 and MC-6) fabricated by direct pyrolysis have the basically blocky and rod-like morphology, which can imply that the method of pre-carbonization is closely related with the morphology change of MC. What's more, high-magnification observation (the insets in Fig. 1) reveals the existence of abundant porous structure in all the samples of MC, which may be inherited from the unique microstructure of the plants and attributed to the method of pre-carbonization and KOH activation process.

**Fig. 2:**
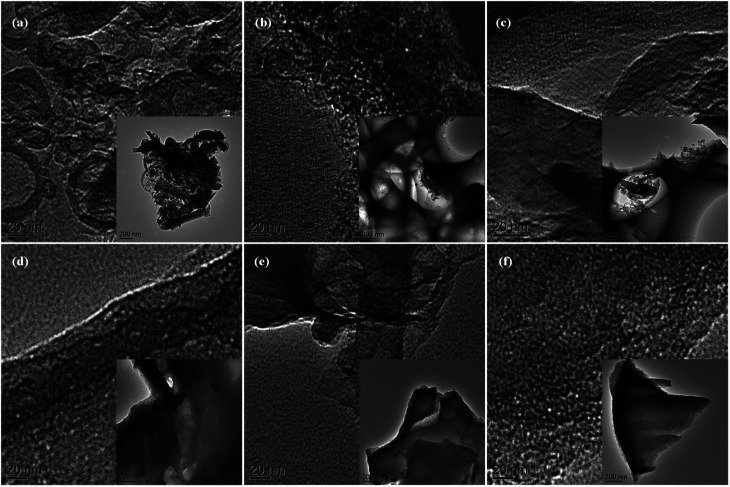
TEM images of MC: (a) MC-1, (b) MC-2, (c) MC-3, (d) MC-4, (e) MC-5, and (f) MC-6. The insets are TEM images under low magnification.

The detailed porous nanostructure of MCs has been further studied by TEM technique (Fig. 2). The TEM images (Fig. 2a–f) again indicate that the MCs with hierarchical porosity structures have been successfully produced. Numerous micropores and mesopores in all the samples can be clearly observed. Furthermore, both amorphous carbon and graphitic structure are identified in the TEM images.

**Fig. 3:**
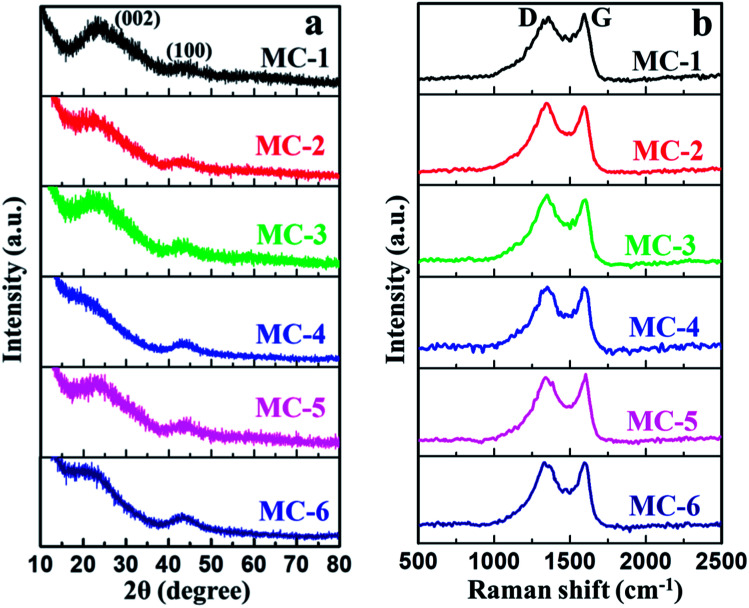
(a) XRD patterns and (b) Raman spectra of MC.

The structures of MC were well investigated by X-ray diffraction (XRD) patterns and Raman spectra. The XRD patterns of MC were collected and the results are shown in Fig. 3a. As can be seen, all the samples of MC show two characteristic diffraction peaks. The weak diffraction peaks of the sample around 44° can be attributed to the (100) diffraction peak of a graphitic-type carbon structure with a limited degree of graphitization of MC.^51^ The broad (002) diffraction peaks usually located at about 23° suggests a disturbed structure in the amorphous state of all the samples of MC due to the randomly oriented aromatic carbon sheets, which is beneficial for creating high specific surface area (SSA).^52^ It is noted that the broad (002) diffraction peak positions of all the samples of MC show slightly different. The difference of the (002) diffraction peak positions may be attributed to KOH activation process and the different surface doping amount of N and O heteroatoms of MC derived from different moxa floss (Table S2, see ESI†). In general, KOH activation can lead to expansion of the carbon structure, a breakdown of aligned structural domains and a random distribution of aromatic carbon sheets.^53^ Furthermore, the incorporation of heteroatoms in the carbon lattice can lead to amorphization of the graphitic carbon and a distorted carbon structure with relatively large interplanar spacing.^54^ These bent structures with long interlayer distances may be able to significantly enhance the electrochemical performance of MC. The Raman spectra (Fig. 3b) of MC clearly exhibit the existence of two intensive bands at around 1348 cm^−1^ (D band) and 1598 cm^−1^ (G band). The D band is associated with the defects and impurities in disordered carbon structure, while the G band arising from the bond stretching of all sp^2^-bonded pairs is should be attributed to graphite in-plane vibrations.^55^ The intensity ratio of the D and G band (*I*_D_/*I*_G_) is well known to be an indicator of the disorder degree of MC. The *I*_D_/*I*_G_ peak ratios of all the samples of MC are around 1.00, exhibiting a relatively low degree of graphitization and containing a large number of disordered carbon structures.^56^

**Fig. 4:**
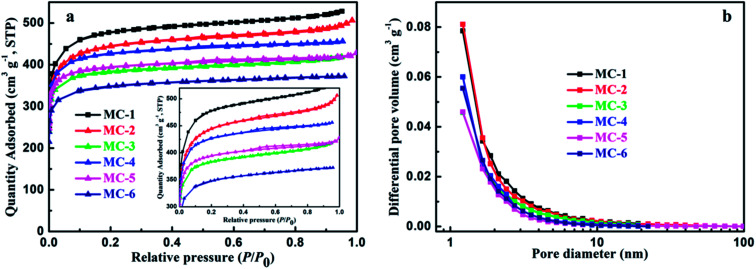
(a) Nitrogen adsorption–desorption isotherms and (b) pore size distribution (PSD) of MCs.

To further evaluate the pore properties of MC, nitrogen absorption–desorption isothermal analysis were employed. As shown in Fig. 4a, it is obvious that two samples of MC exhibit a type I isotherm with H4 hysteresis loops typical for narrow slit-like pores according to the IUPAC classification, that is to say, the isotherms present a steep increase at the very low relative pressure, which can be attributed to the existence of large numbers of micropores,^51^ and then keep almost horizontal with increasing the relative pressure. The distinct hysteresis loops (the inset of Fig. 4a) at the middle relative pressure (*P*/*P*_0_ = 0.4–0.8) prove the presence of abundant mesopores in all the samples of MC.^52^ Moreover, the isotherms rise slightly near the saturated vapor pressure, which demonstrates the existence of macropores.^51^ The features of these isotherms indicate that the samples of MC exhibit a well-developed hierarchical porous structures including the micro-, meso-, and macro-pores. The pore size distributions (PSD) of all the MC samples (estimated by the BJH method) are clearly shown in Fig. 4b. As can be seen, it is obvious that MC has well-developed micropores with pore width less than 2 nm, considerable mesopores (2–50 nm) and the existence of macropores (>50 nm). The SSA and pore structure parameters of MC are summarized in Table S1.† The samples of MC (MC-1, MC-2 and MC-3) by hydrothermal carbonization exhibit high SSA in the range 1472.5–1788.6 m^2^ g^−1^ with the total pore volume ranging from 0.6476 to 0.8170 cm^3^ g^−1^ and the average pore sizes are 1.8271 nm, 1.9391 nm and 1.7591 nm, respectively, while the samples (MC-4, MC-5 and MC-6) by direct pyrolysis possess high SSA in the range 1323.0–1628.0 m^2^ g^−1^ with the total pore volume ranging from 0.5761 to 0.7058 cm^3^ g^−1^ and the average pore sizes are 1.7342 nm, 1.8520 nm and 1.7418 nm, respectively. Clearly, as the storage years of moxa floss increase from 0 year to 5 years, the BET SSA and pore volume of MCs decrease. Furthermore, the MC by hydrothermal carbonization possesses the higher BET SSA, larger pore volume and the longer average pore size than that of MC derived from the same storage years by direct pyrolysis.

### 3.2 Chemical composition analysis of MC

**Fig. 5:**
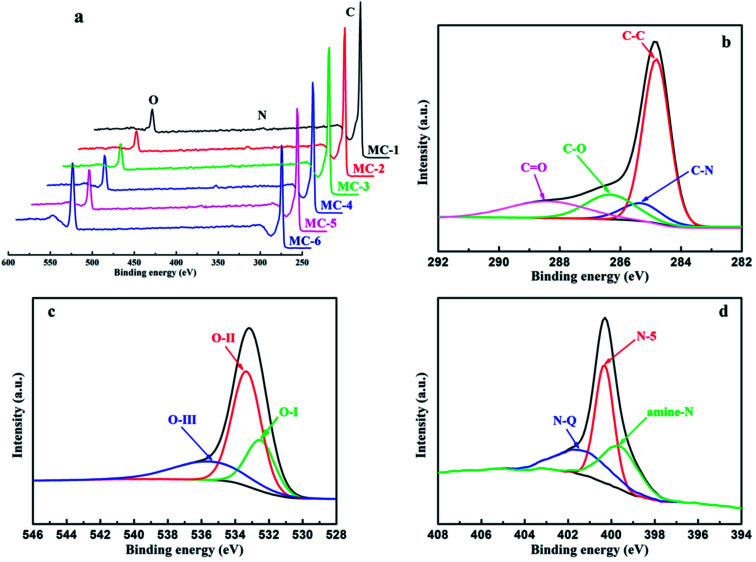
(a) XPS spectra of MC; the typical high-resolution XPS spectra from MC-1: (b) C1s, (c) O1s, (d) N1s.

X-Ray photoelectron spectroscopy (XPS) was performed to investigate the surface chemical compositions and analyze the surface functional groups of MC. As shown in Fig. 5 and Table S2,† all the samples of MC mainly consist of C, N, and O elements. Furthermore, as the storage years of moxa floss increase from 0 year to 5 years, the C atomic concentration in MC by hydrothermal carbonization reduced from 91.72% to 90.62%, the N atomic concentration in MC-2 is the highest and the O atomic concentration in MC-2 is lowest among MC-1, MC-2 and MC-3, while the C atomic concentration in MC by direct pyrolysis reduced from 88.58% to 75.02% and the content is lower than that in MC by hydrothermal carbonization, the N atomic concentration in MC-5 is the highest among MC-4, MC-5 and MC-6 and the O atomic concentration increases from 9.68% to 23.90% and the content is higher than that in MC by hydrothermal carbonization. The significant changes in atomic concentration indicate that the storage years of moxa floss and pre-carbonization can effectively regulate the surface composition of MC. The fitted high-resolution C1s, O1s and N1s spectra of MC-1 are shown in Fig. 5b–d, and other samples are depicted in Fig. S1.† The high-resolution C1s spectra of MC can be resolved into four individual component peaks standing for four different types of carbon functional groups: C–C/C=C (centered at 284.8 eV), C–N (centered at 285.3 eV), C–O (centered at 286.3 eV), and C=O (centered at 288.4 eV). The high-resolution O1s spectra of MC clearly present the existence of several oxygen-based groups including three peaks centered at 532.5, 533.3 and 535.6 eV, representing C=O quinone type groups (O–I), C–OH phenol groups and/or C–O–C ether groups (O-II), and chemisorbed oxygen (COOH carboxylic groups) and/or water (O-III), respectively. Moreover, the high-resolution N1s spectra of MC can be deconvoluted into three peaks, which are assigned to amine nitrogen (amine N) located at around 399.6 eV, pyrrolic/pyridine (N-5) located at around 400.3 eV, and quaternary (N-Q) located at around 401.3 eV. These results indicate that there are numerous heteroatoms doped in the surface of MC and these N and O functional groups can improve pseudocapacitance through faradaic reactions and enhance electrical conductivity. Therefore, with controllable heteroatoms (N and O) doping state, two different morphologies, high specific surface areas, and hierarchical porous structure, all the samples of MC are expected to exhibit excellent electrical conductivity.

### 3.3 Electrochemical characterization of MC

**Fig. 6:**
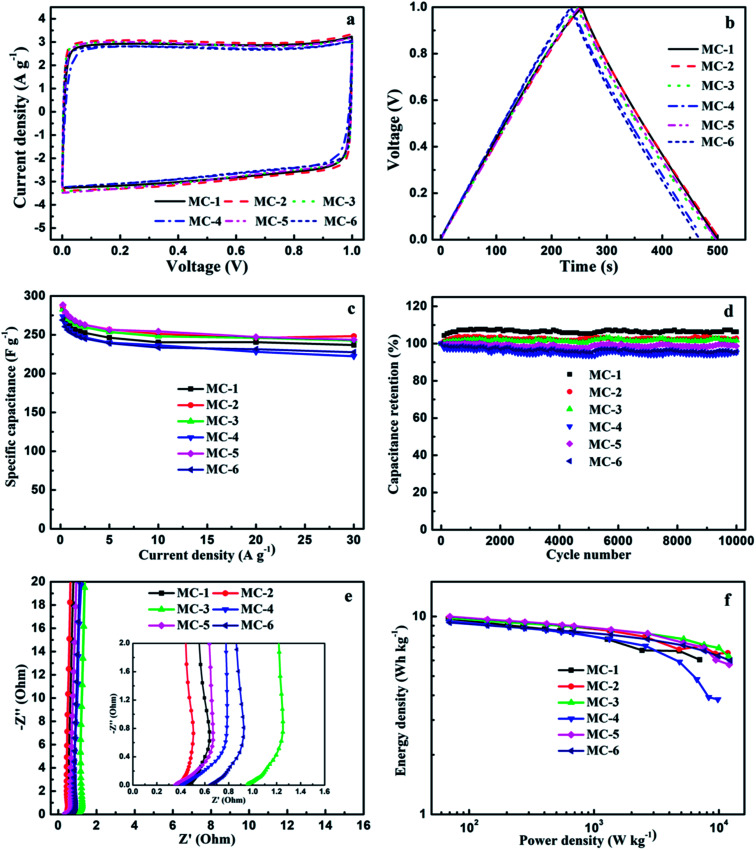
(a) Cyclic voltammetry curves of MC supercapacitors with 6 mol L^−1^ KOH aqueous solution electrolyte from 0 to 1 V at a scan rate of 25 mV s^−1^, (b) galvanostatic charge–discharge curves at a current density of 0.5 A g^−1^, (c) The specific capacitance of MC at different current densities from 0.25 to 30 A g^−1^, (d) cycling stability of MC at a current density of 5 A g^−1^ over 10 000 cycles, (e) electrochemical impedance spectra (Nyquist plots) and (f) Ragone plots of MC supercapacitors in the 6 mol L^−1^ KOH aqueous solution.

The electrochemical performances of MC as the electrode materials for supercapacitors were evaluated by conducting the cyclic voltammetry (CV), galvanostatic charge–discharge (GCD), and electrochemical impedance spectroscopy (EIS) tests for a traditional symmetric two-electrode system in the 6 mol L^−1^ KOH aqueous solution electrolyte at room temperature (Fig. 6).

The typical CV curves (Fig. 6a) of all samples of MC at a scan rate of 25 mV s^−1^ in the potential range from 0 to 1 V show a nearly symmetrical rectangular shape in their CV curves, indicating an ideal double-layer capacitance behavior based on ionic adsorption and exchange, although their morphologies are different; and with the scan rate increasing from 5 to 200 mV s^−1^, they still remain a rectangular-like shape (Fig. S2a†), suggesting that these MC electrodes have an excellent high-rate capacitive behavior and a quick charge and discharge feature,^57^ which may be attributed to the N and O functional groups and hierarchical porous structure (the balanced micropores and mesopores) of MC. These results demonstrate that the MC with two different morphologies (the porous nanosheet morphology and the blocky and rod-like morphology), both can exhibit excellent capacitive performance.

The GCD curves (Fig. 6b) of these MC electrodes at a current density of 0.5 A g^−1^ are liner and show a nearly perfect isosceles triangle shape with negligible IR drop. Similarly, the GCD curves of these MC electrodes at different current densities also exhibit a nearly perfect isosceles triangle shape shown in Fig. S2b.† More importantly, the GCD curves still maintain triangle shapes at a high current density of 30 A g^−1^. These results indicate that the MC electrodes in KOH aqueous solution electrolyte have good charge–discharge property and good capacitive behavior contributed by the pseudocapacitance of the N and O functional groups and abundant micropores, which are corresponding to the CV results. The good charge–discharge property may be associated with the large effective surface areas which can guarantee ion storage space, large numbers of mesopores which can improve easily ion diffusion channels. The specific capacitance (*C*_s_) values of MC from the discharge curves at a constant current density of 0.5 A g^−1^ shown in Fig. 6b are 271.0 F g^−1^ for MC-1, 278.0 F g^−1^ for MC-2, 274.5 F g^−1^ for MC-3, 263.8 F g^−1^ for MC-4, 279.3 F g^−1^ for MC-5, and 260.5 F g^−1^ for MC-6, respectively.

The specific capacitances (*C*_s_) of the MC electrodes with current densities ranging from 0.25 to 30 A g^−1^ calculated from the discharge curves after IR drop in the GCD tests (Fig. S2b†) are shown in Fig. 6c. As can be seen, *C*_s_ decreases normally with the current density increasing from 0.25 to 30 A g^−1^. It is noteworthy that even though the current density is increased up to 30 A g^−1^, the MC electrodes retain an excellent capacitance performance with 81.3–86.7% retention of its initial specific capacitance at a current of 0.25 A g^−1^, indicating excellent rate capability. The excellent rate capability and high specific capacitance retention of these MC electrodes at high current density are most likely due to its large-size multiscale nanoarchitecture, high surface area for fast ions transport-charge storage, and the coexistence of nitrogen- and oxygen-containing functional groups, which can also make the surface of carbon materials more easily infiltrated by electrolytes and deliver ultrafast electron transport path even at high current density and thus enhance their capacitance. Furthermore, the specific capacitances (*C*_s_) of these MC electrodes are comparable or even higher than that of most recently reported biomass-derived carbons for supercapacitors (Table S3†).

For supercapacitors in practical applications, the cycling stability is one of the most important parameters and counts for a great deal. Therefore, the cycling stability of supercapacitors based on MC was investigated in a two-electrode configuration at a current density of 5 A g^−1^. As can be seen in Fig. 6d, all samples of MC show above 95.1% capacitance retention of their initial capacitance even suffered from 10 000 charge–discharge cycles. The result indicates that MC has excellent electrochemical cycling stability.

To further explore the capacitive behaviors of MC, electrochemical impedance spectroscopy (EIS) tests were carried out with the amplitude of 5 mV in the frequency range of 10^5^ to 10^−3^ Hz referring to open circuit potential. The Nyquist plots curves of the MC electrodes are shown in Fig. 6e. Clearly, in the low frequency region, the Nyquist plots of MC exhibit almost vertical curves, suggesting an ideal electric double layer capacitive behavior. Besides, Nyquist plots show a Warburg resistance section in medium frequency region with a slope of about 45°. The equivalent serial internal resistances of the MC-based supercapacitors are about 0.45 Ω for MC-1, 0.36 Ω for MC-2, 0.95 Ω for MC-3, 0.41 Ω for MC-4, 0.35 Ω for MC-5 and 0.64 Ω for MC-6, respectively, obtained from the real axis intercept of the Nyquist plots in the high frequency region, indicating the excellent electrical conductivity of MC. The excellent electrical conductivity of MC can be ascribed to the cooperation of the high specific surface area (SSA), hierarchical porous structure and heteroatom (N and O) functional groups which can provide highly efficient charge transfer and ion diffusion channels, and thus lead to the MC electrodes with the excellent rate performance. The intercepts of the MC-2 and MC-5 electrodes are shorter than that of others, suggesting their lower equivalent serial internal resistances, which are consistent with the higher *C*_s_ results of them. This demonstrates that although the morphologies are different, MC obtained from three storage years exhibits faster electrolyte ion reversible diffusion,^58^ and better capacitance performance.

The Ragone plots of the MC supercapacitors are shown in Fig. 6f. The maximum energy density for these supercapacitors is about 10.0 W h kg^−1^ at a power density of 70.3 W kg^−1^, which is higher when compared with that of other recently reported carbon-based supercapacitors.^25,27,59,60^ In addition, the energy density decreases slowly with the rapid increase of power density. Even if the power density increased up to 12.3 kW kg^−1^, the energy density can still retain a value of 6.0 W h kg^−1^, suggesting the superior ability for energy storage of the MC supercapacitors.

**Fig. 7:**
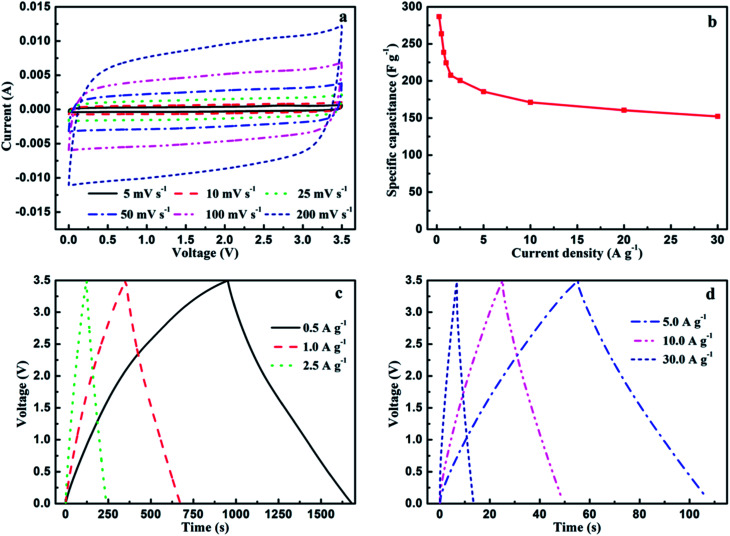
(a) Cyclic voltammetry curves of MC-2 supercapacitors in EMIMBF_4_ electrolytes from 0 to 3.5 V at different scan rates from 5 to 200 mV s^−1^, (b) the specific capacitance of MC-2 at different current densities from 0.25 to 30 A g^−1^, (c) galvanostatic charge–discharge curves at different current densities from 0.5 to 2.5 A g^−1^, (d) galvanostatic charge–discharge curves at different current densities from 5.0 to 30.0 A g^−1^.

MC-2 exhibits higher specific surface area, more balanced micropores and mesopores as well as higher N atomic concentration, which may endow it delivering higher capacitance performance in ionic liquid electrolytes. In order to further evaluate the electrochemical performance of MC-2, supercapacitors based on MC-2 was assembled in the organic electrolytes. More practically, the potential window of ionic liquid EMIMBF_4_ electrolyte is 3.5 times higher than 6 mol L^−1^ KOH. As shown in Fig. 7a, the CV curves of MC-2 still retain a rectangular-like shape without drastic change over a wide range of scan rate increasing from 5 to 200 mV s^−1^, suggesting an ideal electrochemical capacitor. Besides, the GCD curves of MC-2 display a nearly isosceles triangle shape at different current densities as shown in Fig. 7c and d. More importantly, the GCD curves still maintain triangle shape with unobvious IR drop at a high current density of 30 A g^−1^, implying typical electrochemical capacitive behavior, the high charge–discharge efficiency and low equivalent series resistance. The specific capacitances (*C*_s_) of MC-2 electrode with current densities ranging from 0.25 to 30 A g^−1^ are shown in Fig. 7b. As can be seen, *C*_s_ displays a decreasing tendency with the current density increasing from 0.25 to 30 A g^−1^. It is noteworthy that MC-2 exhibits good performance with a high specific capacitance of 286.8 F g^−1^ at a current density of 0.25 A g^−1^ and medium retention rate (152.3 F g^−1^) at a high current density of 30 A g^−1^ (53% of the capacitance retention).

The electrochemical results indicate that although the morphologies of MCs derived from moxa floss of the same storage year by two different pre-carbonization strategies are different, they present excellent performances for supercapacitors application, which can be attributed to their high specific surface areas (high SSA), unique hierarchical porous structures, numerous heteroatom (O and N) functional groups, and good electrical conductivities. High SSA provides sufficient electrode–electrolyte interface to form more electric double layers for charge accommodation; the hierarchical porous structure ensures the rapid ion diffusion by shortening the diffusion pathways, and good electrical conductivity facilitates efficient transport of the electrons, which are important for achieving high-rate performances.^27^ Furthermore, the presence of N and O functional groups makes the surface of MCs more easily infiltrated by electrolytes and enhances their electrical conductivity, and thus further improves the electron and ion transportation efficiency.

## 4. Conclusions

In summary, we have successfully developed a new concept of moxa floss use, that is, the utilization of moxa floss is to fabricate biomass-derived porous carbon for supercapacitors with high performance. Novel MC has been successfully prepared by two low-cost, safe, and facile approaches: hydrothermal carbonization-KOH activation and pyrolysis-KOH activation. Although moxa floss of different storage years were utilized as carbon precursors, the as-obtained MC samples, despite the different morphologies, possess similar physicochemical properties, such as high specific surface area up to 1788.6 m^2^ g^−1^ providing sufficient active sites, abundant hierarchical pores coexisting of micro-, meso-, and macropores, numerous nitrogen- and oxygen- based functional groups, which facilitate electrolyte diffusion and ion transfer, and endow the MC electrodes with good electrical conductivity. Moreover, the as-assembled MC supercapacitors with aqueous electrolyte in a two-electrode system exhibit an outstanding specific capacitance of 288.3 F g^−1^ at 0.25 A g^−1^, an excellent rate performance, and an outstanding long-term cycling stability with 98.7% of initial capacitance retention even suffered from 10 000 charge–discharge cycles at 5 A g^−1^. Therefore, utilizing moxa floss as carbon precursor to prepare biomass-derived activated carbon for supercapacitors is an extremely promising new application of moxa. Meanwhile, in consideration of its renewability, low-cost and the abundance, moxa floss as a novel biomass source significantly responds to the demand of the sustainable development of the society, achieving low-cost and high-performance electrode materials from renewable sources. Considering the fact that the method, time and temperature of the pre-carbonization, KOH activation time and temperature, and the weight ratio of the pre-carbonized product to KOH, play important roles in the regulation of the capacitance performance of the final porous carbons, we will systematically explore the synthesis conditions for morphology change, specific surface area, heteroatom doping level, and the porous structures as well as the correlation between these factors and electrochemical performances of the prepared MCs in our following research.

## Conflicts of interest

There are no conflicts to declare.

## Supplementary Material

RA-008-C8RA01672K-s001
