# Supplementary material for: Heteroatom-doped porous carbons derived from moxa floss of different storage years for supercapacitors
Source: RSC Adv. 2018 May 3;8(30):16433–43. doi: 10.1039/c8ra01672k (PMC9080236; doi:10.1039/c8ra01672k)
Supplement: RA-008-C8RA01672K-s001 [file RA-008-C8RA01672K-s001.pdf]

Electronic Supplementary Material (ESI) for RSC Advances.
This journal is © The Royal Society of Chemistry 2018

Supporting Information

Heteroatom-doped porous carbons derived from Moxa floss of different storage years for supercapacitors

Xuelin Zhang,[a] Qingyuan Niu,[b] Yaqing Guo,[b] Xiyan Gao*[a] and Kezheng Gao*[b]

[a]College of Acupuncture-Moxibustion and Tuina, Henan University of Traditional Chinese Medicine, Zhengzhou 450046, P. R. China
[b]State Laboratory of Surface and Interface Science and Technology, School of Material and Chemical Engineering, Zhengzhou University of Light Industry, Zhengzhou 450002, P. R. China

*Corresponding author:
Xiyan Gao: Tel: +86-371-6593-4802, FAX: +86-371-6593-4802, Email: gaoxiyan@yeah.net
Kezheng Gao: Tel: +86-371-8656-9857, FAX: +86-371-8656-9857, Email: gaokezheng@126.com

Table S1 The SSA and pore structure parameters of MC.

Products	BET SSA ($m^2 g^{-1}$)	Pore volume (V_{total}, $cm^3 g^{-1}$)	Average pore width (nm)
MC-1	1788.6	0.8170	1.8271
MC-2	1616.0	0.7834	1.9391
MC-3	1472.5	0.6476	1.7591
MC-4	1628.0	0.7058	1.7342
MC-5	1420.6	0.6578	1.8520
MC-6	1323.0	0.5761	1.7418

Table S2 Elemental analysis results of MC.

Products	C (wt.%)	N (wt.%)	O (wt.%)
MC-1	91.72	1.58	6.70
MC-2	91.45	2.32	6.23
MC-3	90.62	1.62	7.76
MC-4	88.58	1.74	9.68
MC-5	86.25	1.75	12.00
MC-6	75.02	1.08	23.90

MC-2:

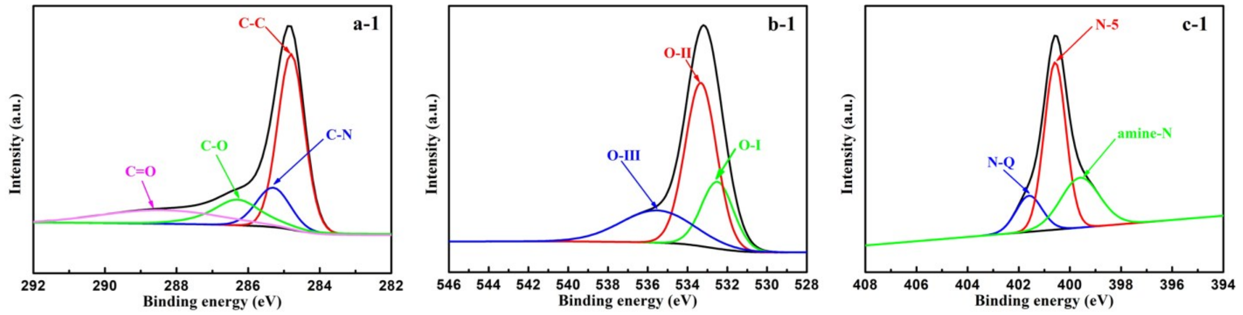

MC-3:

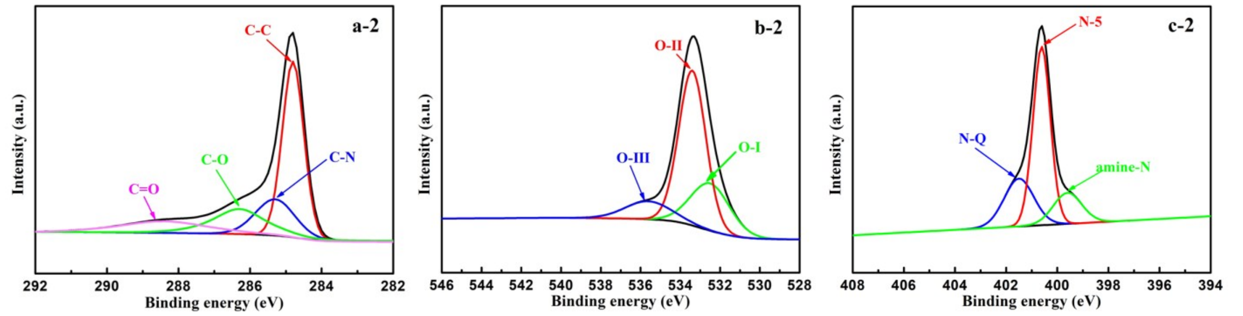

MC-4:

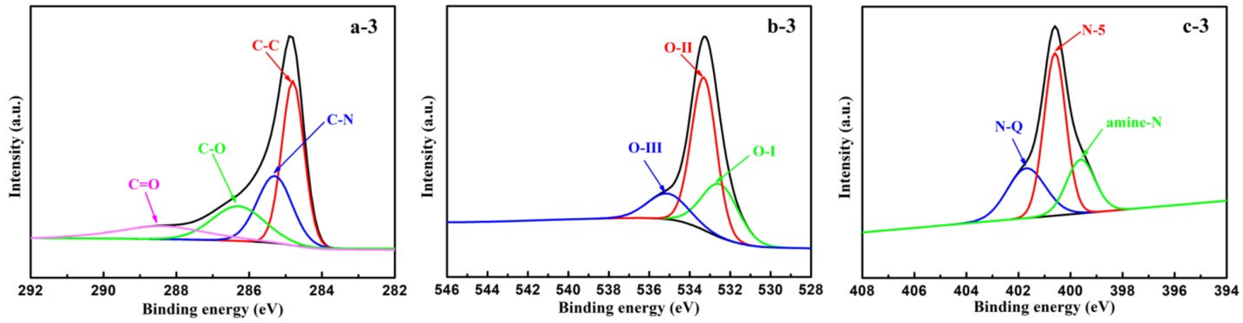

MC-5:

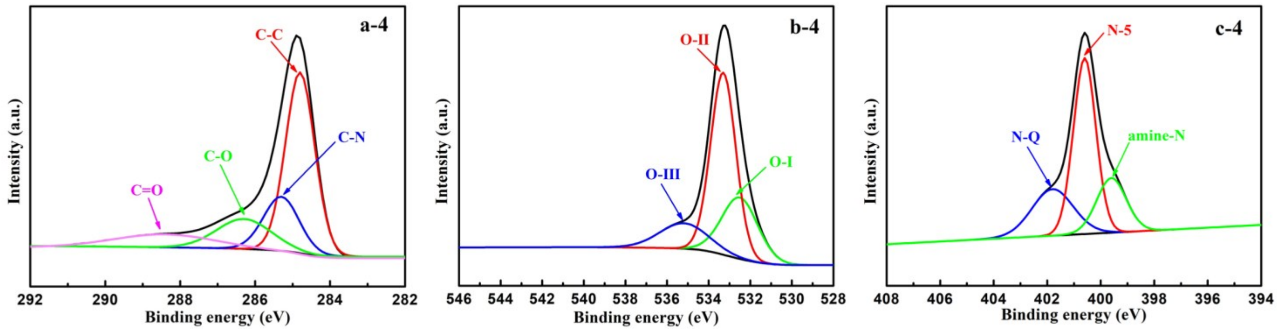

MC-6:

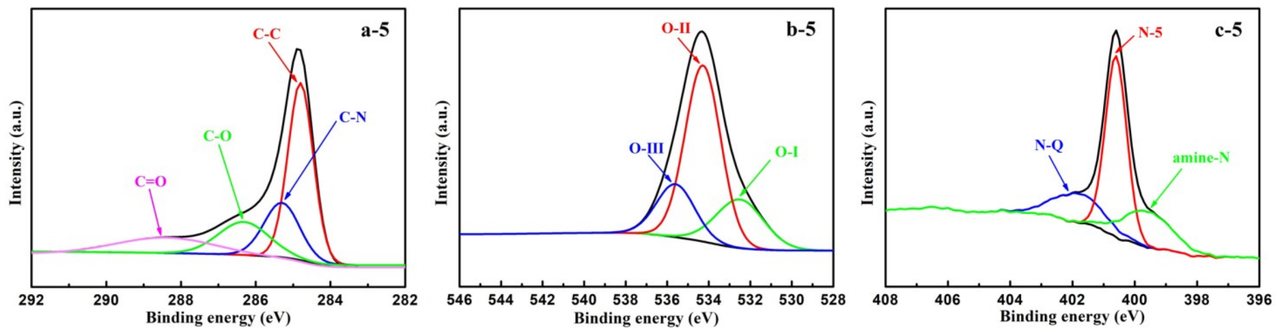

Figure S1 The high-resolution XPS spectra of MC2-6: (a-1~5) C1s, (b-1~5) O1s, (c-1~5) N1s.

MC-1:

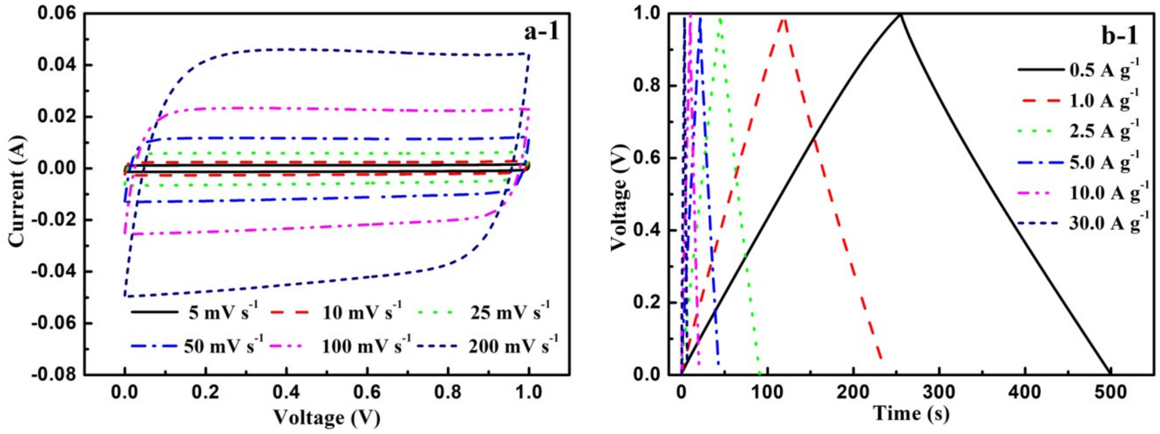

MC-2:

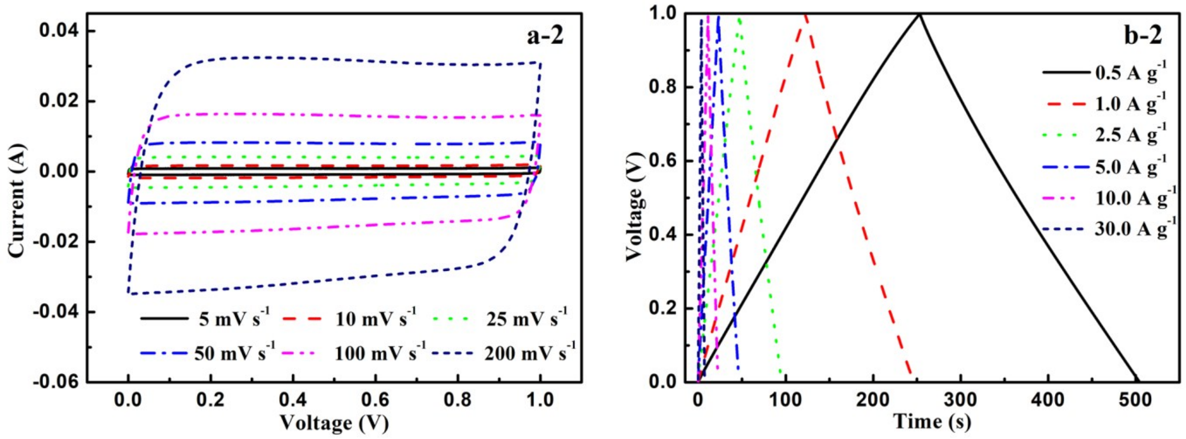

MC-3:

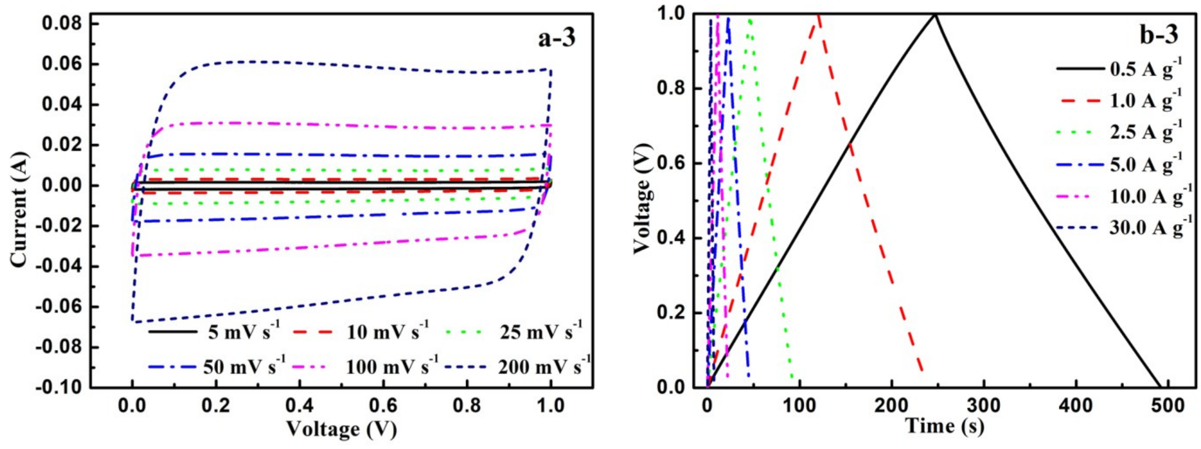

MC-4:

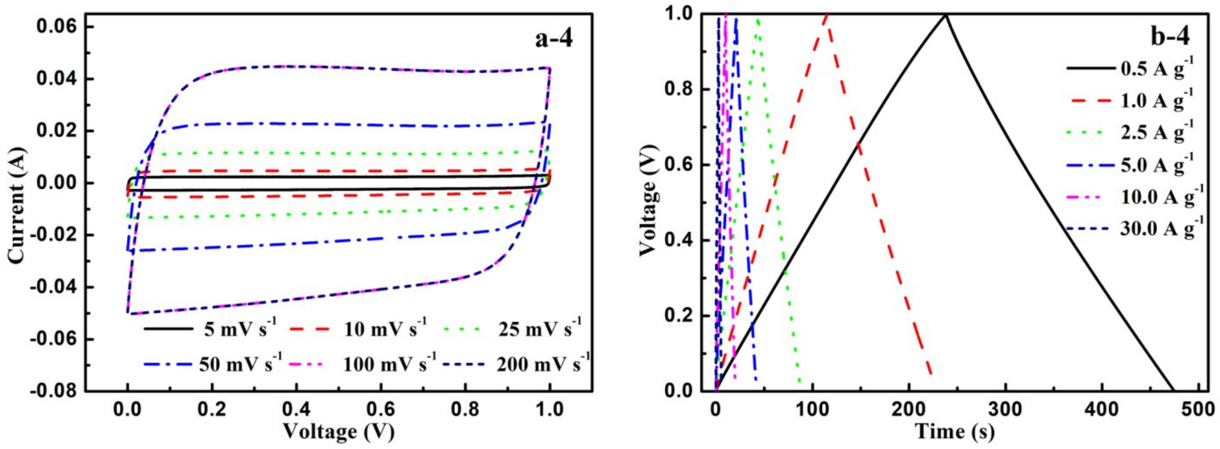

MC-5:

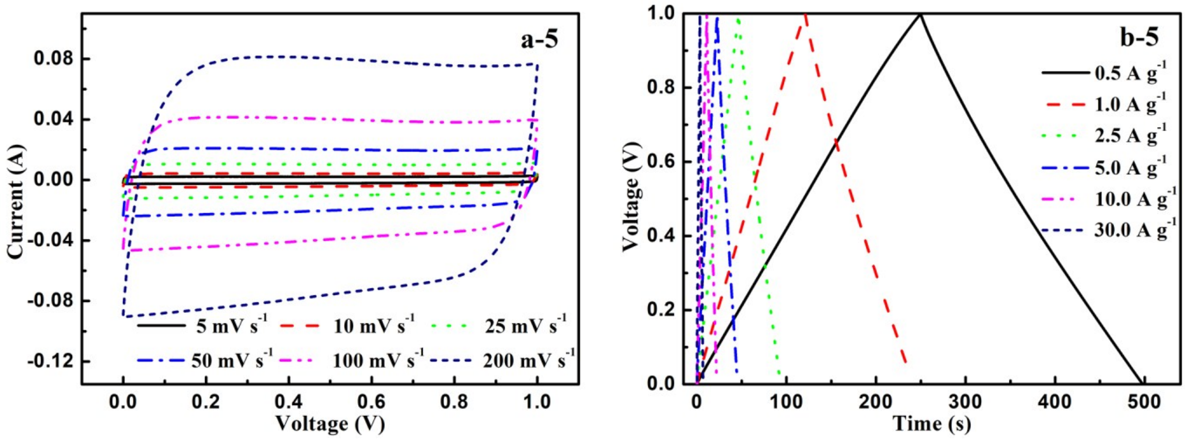

MC-6:

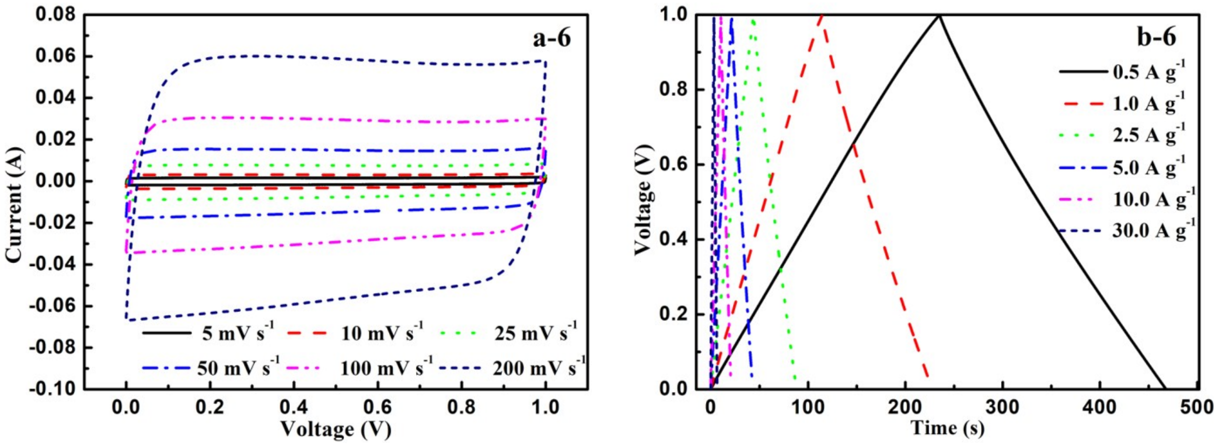

Figure S2 (a-1~6) cyclic voltammetry curves at different scan rates from 5 to 200 mV s^{-1}, (b-1~6) Galvanostatic charge-discharge curves at different current densities from 0.5 to 30.0 A g^{-1} in a two-electrode system with 6 mol L^{-1} KOH aqueous solution electrolyte.

Table S3 comparison of biomass-derived carbons as electrode materials of supercapacitors, all tested in a two-electrode configuration.

Carbon source	SSA ($m^2 g^{-1}$)	Capacitance ($F g^{-1}$)	Electrolyte	Measure condition	Ref.
moxa floss	1616	288.3	6 M KOH	0.25 A g^{-1}	Our work
banana peel	1650	206	6 M KOH	1 A g^{-1}	1
waste tobacco	1104	170	6 M KOH	0.5 A g^{-1}	2
tobacco rods	1761	237	6 M KOH	0.5 A g^{-1}	3
broad bean shells	655	202	6 M KOH	0.5 A g^{-1}	4
coconut shell	1874	268	6 M KOH	1.0 A g^{-1}	5
puffed rice	3326	334	6 M KOH	0.5 A g^{-1}	6
tobacco stem	1749	141	$TEABF_4$/AN	0.2 A g^{-1}	7
cornstalk pith	805	116	6 M KOH	0.25 A g^{-1}	8

References

1. Y. Lv, L. Gan, M. Liu, W. Xiong, Z. Xu, D. Zhu and D. S. Wright, *J. Power Sources*, 2012, 209, 152-157.
2. Y. F. Sha, J. Y. Lou, S. Z. Bai, D. Wu, B. Z. Liu and Y. Ling, *Mater. Res. Bull.* 2015, 64, 327-332.
3. Y. Q. Zhao, M. Lu, P. Y. Tao, Y. J. Zhang, X. T. Gong, Z. Yang, G. Q. Zhang and H. L. Li, *J. Power Sources*, 2016, 307, 391-400.
4. G. Y. Xu, J. P. Han, B. Ding, P. Nie, J. Pan, H. Dou, H. S. Li and X. G. Zhang, *Green Chem.*,2015, 17, 1668-1674.
5. L. Sun, C. G. Tian, M. T. Li, X. Y. Meng, L. Wang, R. H. Wang, J. Yin and H. G. Fu, *J. Mater. Chem. A*, 2013, 1, 6462-6470.
6. J. H. Hou, K. Jiang, M. Tahir, X. G. Wu, F. Idrees, M. Shen and C. B. Cao, *J. Power Sources*, 2017, 371, 148-155.
7. P. Kleszyk, P. Ratajczak, P. Skowron, J. Jagiello, Q. Abbas, E. Frackowiak and F. Béguin, *Carbon*, 2015, 81, 148-157.

8. K. Z. Gao, Q. Y. Niu, Q. H. Tang, Y. Q. Guo and L. Z. Wang, *J. Electron. Mater.*, 2018, 47, 337-346.
